# CellMAP: an open-source software tool to batch-process cell topography and stiffness maps collected with an atomic force microscope

**DOI:** 10.1186/s12859-025-06060-0

**Published:** 2025-02-04

**Authors:** Antoine Allard, Maxime Liboz, Raphaël Crépin, Sid Labdi, Olek Maciejak, Michel Malo, Clément Campillo, Guillaume Lamour

**Affiliations:** 1 LAMBE, CNRS, Université Paris-Saclay, Univ Evry, CY Cergy Paris Université, 91025 Evry-Courcouronnes, France; 2 LOMA, UMR 5798, CNRS, Universite de Bordeaux, Talence, France; 3 Institut Universitaire de France (IUF), 75231 Paris, France

**Keywords:** Cell mechanics, Topography, Atomic force microscopy, MATLAB, Graphical user interface, Batch processing

## Abstract

Atomic force microscopy (AFM) is the gold-standard technique to simultaneously map the morphology and viscoelastic properties of living cells. Although existing software tools, both open-source and from AFM manufacturers, can analyze cells individually, there is a growing need for fast and accessible codes to compile data from multiple cells into a single dataset. To address this, we present CellMAP, a user-friendly software tool that streamlines the batch-processing of AFM-derived topography and stiffness maps of living cells. Our analysis pipeline includes but is not limited to: flattening of the underlying substrate surface, filtering of outlier values, measurement of the cell surface and volume, and measurement of height and stiffness distributions. CellMAP can also generate a composite cell that reflects the height and stiffness properties of an entire cell population.

## Background

Atomic force microscopy (AFM) is a powerful technique to characterize living cells in physiological conditions of temperature and pressure and without incorporating external probes such as fluorescent dyes in the cells. AFM quantifies the interaction between a sample surface and an external probe, typically a stiff nanometer-sized tip mounted on a flexible cantilever [1]. In tapping mode, the amplitude and frequency of the cantilever’s vibration can be monitored to extract the topography and mechanical properties [2]. In force mapping or force-volume modes, the deflection of the cantilever is used to measure the contact force between the sample and the probe as a function of the indentation depth [3]. This enables the recording of force-distance curves collected vertically all across the sample surface, i.e. for every pixel of an image [4]. Processing the force curves with appropriate contact mechanics models thus provides the local height and stiffness properties, such as the Young’s modulus [5–9].

Measuring cell morphology and stiffness helps characterizing not only healthy processes such as cell division and motility [10, 11], but also disease-related processes such as cancer cell migration and infection [12–15]. Furthermore, the search of mechanical biomarkers for diagnosis is an active line of research. For example, monitoring the stiffness and morphology of red blood cells can reveal blood disorders [16]. However, most current methods require manipulation of large datasets that exhibit large variability due to both intra-cell and inter-cell heterogeneity. In addition, AFM itself generates measurement artifacts each time aberrant force-distance curves are recorded, which leads to aberrant height and stiffness values in the resulting maps. To achieve proper analysis, it is therefore critical to clean single AFM images from artifacts, as well as to combine the measurements made on many distinct AFM images. In the example above, a trustworthy diagnosis would thence rely on the ability of the software tool to, first, identify the wrong pixels and take them out, and second, to combine the measurements made on many cells that belong to a single cell population.

Multiple open-source software tools can extract height and stiffness maps from force curves and analyze these maps quantitatively [17–20]. For example, AtomicJ offers batch-processing for force curves with customizable parameters, while Gwyddion provides a wide range of processing functions, including flattening operations and volume calculations. However, these tools are either limited to single-image processing or lack integrated statistical analysis for large-scale datasets. To address these limitations, we developed CellMAP, an open-source software tool designed specifically to handle AFM data for the mechanics and morphology of a batch of many cells and provide statistical outputs for entire cell populations.

**Fig. 1:**
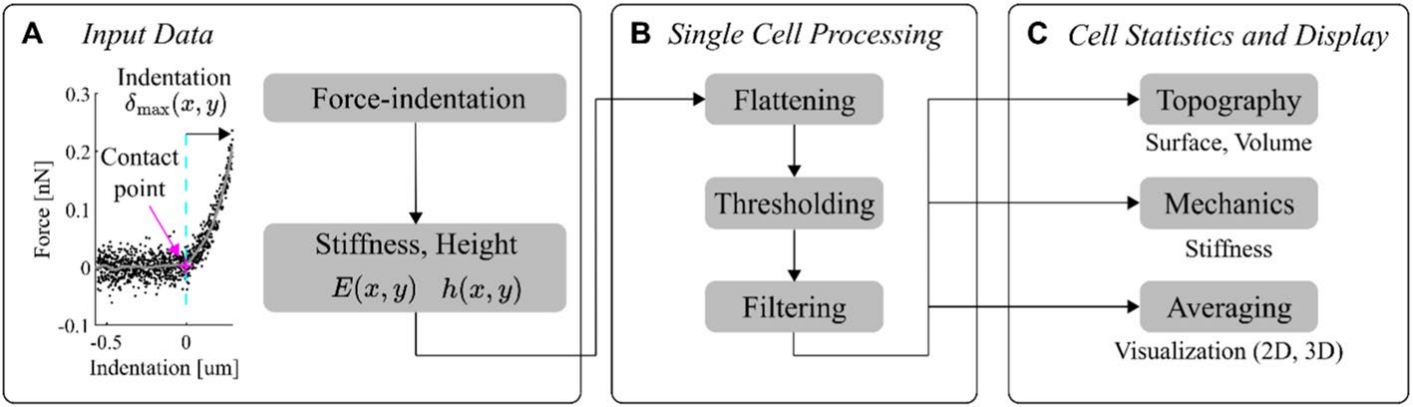
Workflow of the AFM Data Analysis Program CellMAP. **A** CellMAP reads preprocessed 2-dimensional maps (for instance, stiffness $E$ or height $h$) derived from force‒indentation curves analyzed at each map coordinate $(x,y)$. **B** CellMAP processes individual maps via a pipeline that includes flattening (to flatten the background substrate), thresholding (to select data on the basis of stiffness, height, and relative indentation values), and filtering (to smooth isolated points). **C** CellMAP gathers the processed data from multiple cells to estimate the statistical parameters and distributions for cell topography and mechanics

To address the above-mentioned shortcomings in current AFM software (namely, the inability to easily handle artifacts across multiple AFM images, and the lack of tools to process and analyze data across many cells in a batch), we have developed CellMAP whose name stands for *Cell*ular *MA*pping and *P*rocessing from AFM data. CellMAP features a Graphical User Interface (GUI) working under MATLAB or as a standalone application. Figure 1 illustrates CellMAP’s workflow. Using CellMAP without programming knowledge, the user can handle large datasets composed of many images, manipulate these images, derive height and stiffness measurements, and pool the results in the form of histograms or statistical indices related to a single population, as shown in a recent study in which we systematically compared the characteristics of different cell populations cultured on micropatterns [12]. In another recent work, we used CellMAP to monitor changes in cell stiffness and morphology as a function of time [10].

Here, we showcase CellMAP’s capabilities by batch-processing AFM images of human breast cancer cells. We show how CellMAP enables users to filter the data and make measurements on each individual cell, and then to gather the results of multiple cells to quantitatively characterize the cell population as a whole. We verify CellMAP’s performance in calculating surfaces and volumes by using simulated hemispheres of known parameters. Finally, we introduce a graphical tool that allows the user to plot an average cell representing the whole cell population. This tool can be useful if the user collects images of cells or other objects that are morphologically similar in all images, as it is for polarized cells (e.g., myotubes) or for cells cultured on micropatterns whose geometry is constrained [21].

## Implementation

### System requirements

CellMAP requires MATLAB (R2020b or above), or MATLAB Runtime [22]. The MATLAB Runtime is a free software component that enables users to run standalone applications like CellMAP without needing a full MATLAB license. However, users who wish to modify or extend the CellMAP code must have a licensed version of MATLAB, as the MATLAB Runtime does not provide a development environment. CellMAP has been optimized to process AFM maps and manipulate force curve files (*.jpk-qi-data) generated using a Nanowizard 4 AFM (JPK-Bruker) and preprocessed via the JPK Data Processing software tool. However, CellMAP can be used to process any height or stiffness map that has the format of a 2-dimensional matrix saved in a .txt file. Data should be organized as shown in the example folder “Cell”. Within one master folder that corresponds to a cell population, subfolders should be created for each individual cell that belongs to that population. While the presence of force curves is optional, each subfolder should contain at least one map (*.txt).

### Installation

CellMAP, together with test files and source code, can be downloaded online for free [23]. Two types of installations are possible. For MATLAB license owners, run it, click on Apps from the toolbar, “Install App” and select *CellMAP.mlappinstall*. After completion, a new icon called CellMAP is added to the Apps list. To use CellMAP outside of the MATLAB software, it is required to install the MATLAB Runtime [22] and execute the standalone application CellMAP.exe.

### Import/Export

Two options are available in the “File” menu: “New session” and “Load session”. The latter enables the user to reload a previous workflow. Otherwise, click on the “New session” item. Select the *folder* that contains the subfolders you wish to analyze, organized as described above (see System Requirements). All subfolders are also loaded by CellMAP (e.g., a dataset in which each subfolder corresponds to a distinct cell). When force curves are available (e.g., in a force mapping file), a dialog box enables the user to load these force curves.

Immediately after this importation, these data are available under the name “Raw” in the “Process” drop-down button. This button will be subsequently updated depending on the process applied (see Sect. 4.1 Single Cell Processing). At any time, the user can click on “Export” to save the session as it is. A *.dat file is saved and can be reloaded in CellMAP later (“File/Load session”).

### Initial settings

**Fig. 2:**
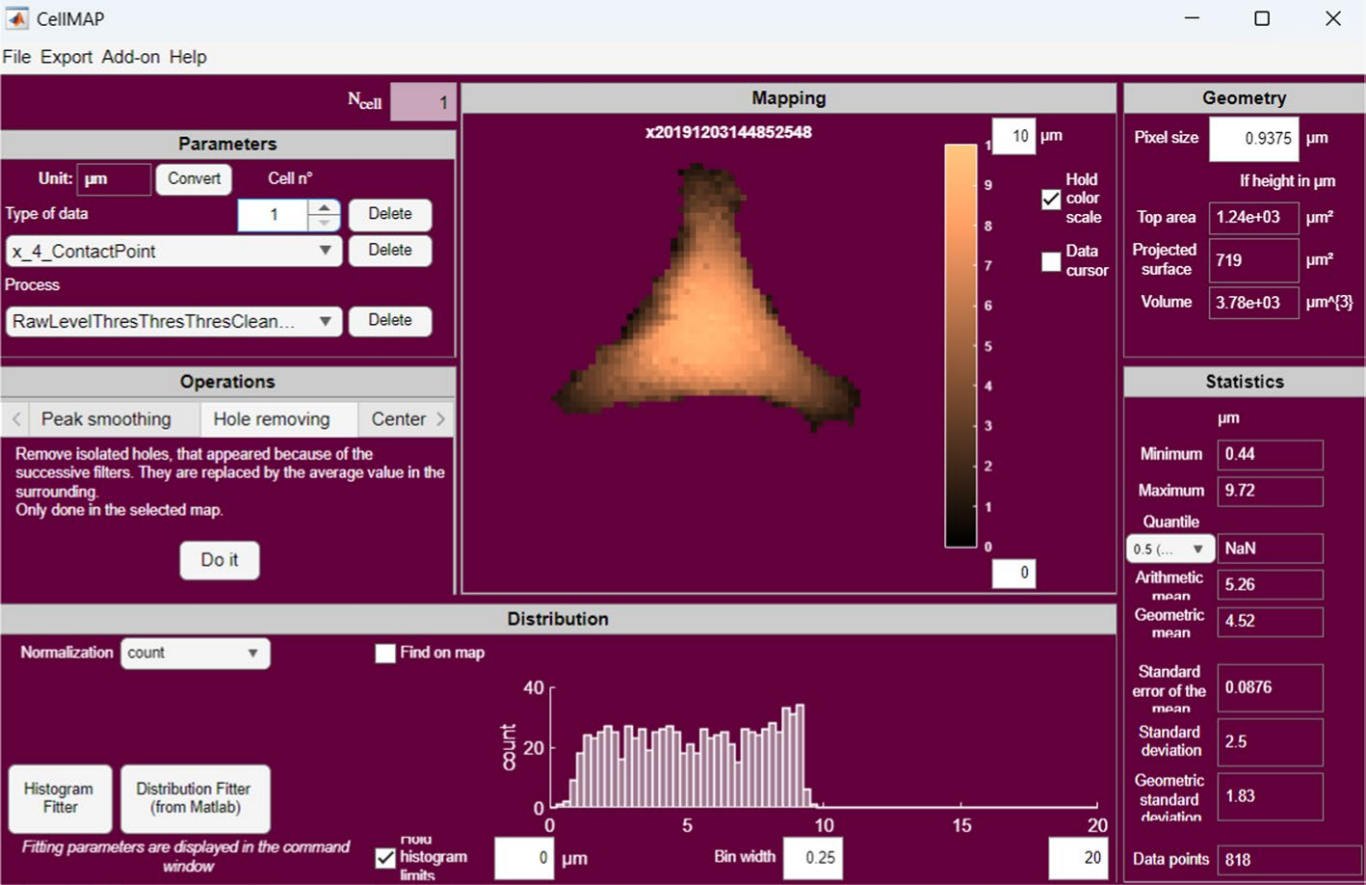
A screenshot of the CellMAP user interface. The interface includes six distinct panels: “Parameters” for adjusting settings, “Operations” for processing data, “Mapping” for representing images, “Distribution” for displaying histograms of the data, “Geometry” for calculating the volume and surface area of the imaged object, and “Statistics” for presenting parameters that characterize the distribution

After completion of the loading step, the data are displayed in the “Parameters” panel (Fig. 2). The procedure we present in this section is a guided tour to handle raw data. AFM maps and their characteristics are automatically updated in the “Mapping”, “Distribution”, “Geometry” and “Statistics” panels. The “Parameters” panel enables the user to select and visualize one dataset. All loaded cells are numbered and accessible via the edit box “Cell n°”. In the example of Fig. 2, at least two “Types of data” are available with the drop-down button (e.g., height, stiffness). Their unit can be changed via the “Convert” button, allowing, for example, the user to convert “kPa” to “Pa” (type “1e3” in the dialog box, 1 kPa = 1 × 10^3^ Pa ). If force curves have been loaded, a new variable called “Indentation” is available, hereafter denoted ${\delta }_{max}$ (see Fig. 1). This quantity represents the highest indentation depth measured in the force curve.

The user can adjust the display of the data shown in the “Mapping” and “Distribution” panels. The color scale of height and stiffness maps can be adjusted via the corresponding edit boxes. If no image is visible, the color bar might not be properly adjusted: values displayed in the “Statistics” panel or looking at the histogram in the “Distribution” panel may guide the choice of appropriate values. Similarly, the settings of the histogram displayed in the graphical user interface are editable using “Bin width” and limit boxes. The “Hold color scale” and “Hold histogram limits” tick buttons enable keeping the boundaries constant while browsing from one cell to another in the “Mapping” and “Distribution” panels, respectively. Otherwise, the parameters are adjusted automatically based on highest and lowest values for each cell. With respect to these histograms, the type of “Normalization” can be specified, whose names (i.e., pdf, cdf, etc.) are formally defined in MATLAB online documentation (see Normalization properties from the *histogram* function).

### Additional functions

“Data cursor” adds the option to display the value at a user-defined coordinate (*x,y*), as well as the horizontal and vertical line profiles around this point. The selection of this point can be changed using (i) the bottom and left sliders or (ii) the arrow from your keyboard (a click on the map might be needed). If previously loaded, the force-indentation curve at this space coordinate is shown in a separate window. We also added the option, called “Find on the map”, to localize spatially all the data points corresponding to a user-defined histogram bin: it is picked by (i) using the top slider or (ii) scrolling with the mouse.

Another useful add-on is the possibility to keep track of your “Pipeline”, as CellMAP records all procedures applied to the dataset that is being used. After completion, “Stop recording” saves this workflow, which can be reloaded to process another dataset in the exact same way. “Data” can be exported as *.txt files, enabling the user to work with processed data outside of CellMAP. Files are then available in the working folder.

## Results

Here, we describe all the CellMAP functions described in Fig. 1. According to their specific needs, users can select the processing functions required for their analysis. In this work, height and elasticity maps derived from force‒indentation curves were preprocessed using the JPK-Bruker Data Processing software. However, CellMAP enables users to work with raw force‒distance curves and run their own preprocessing to (i) detect the contact point via the bottom effect cone correction (BECC) method developed by Chadwick’s group [6], which accounts for artefacts due to stiff substrates in AFM measurements of thin samples, and/or (ii) to fit force‒indentation curves via the model of their choice.

### Single cell processing

Force mapping results in both a height map and a stiffness map for each cell imaged on a flat substrate (Fig. 1A). To ensure the robustness of the quantitative measurements made on each individual cell, it is essential to correct sample tilt and filter out outlier data points. This can be achieved via a sequence of processing operations implemented in the CellMAP code and easily accessible in the “Operations” panel of the CellMAP Graphical User Interface (GUI; Fig. 2). CellMAP processes individual height and stiffness maps via substrate leveling, data thresholding and filtering (see Fig. 1B). At each processing step, the resulting maps are automatically saved, and thence users can easily access any previously processed data, as well as unprocessed “Raw” data. Additionally, a brief “Description” of the processed maps and data is available in the “Help” tab.

#### Flattening

**Fig. 3:**
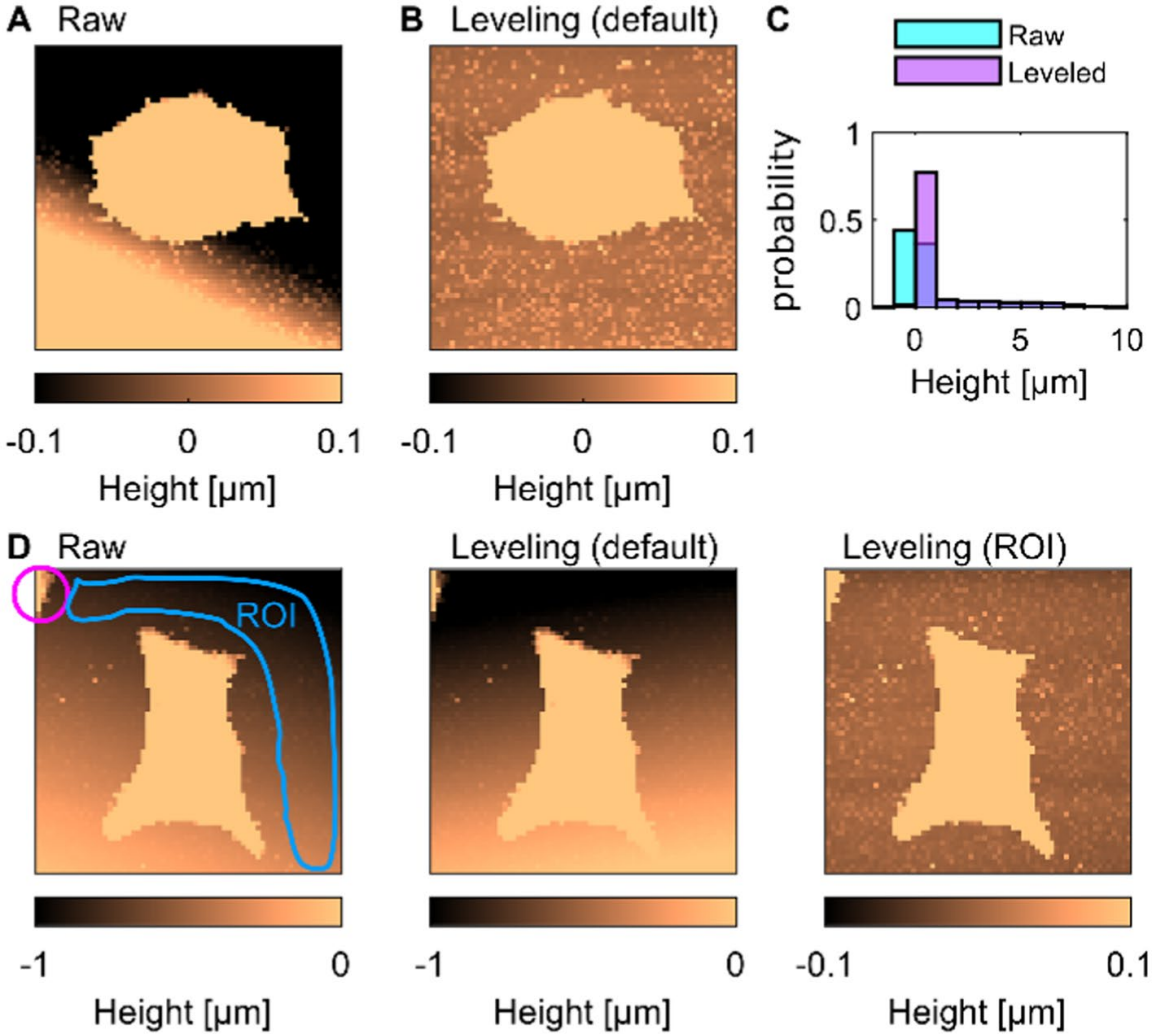
Flattening, or correction of the tilt. **A**–**B** Height maps (**A**) before and (**B**) after the “Flattening” operation. (**C**) Histograms of the cell height in the maps shown in (**A**) and (**B**). **D** Example of a tilt correction using a user-defined Region of Interest (ROI). The default flattening that results in the height map in the middle panel is incorrect because of a defect on the substrate (pink circle in the left panel). Using a user-defined ROI results in correct flattening (right panel)

Flattening consists in correcting the substrate tilt, which is crucial for accurate measurements of the cell height. For example, even a 0*.*1° tilt for a 100 μm × 100 μm image can cause a vertical drift of approximately $200 \text{nm}$ and lead to measurement bias (Fig. 3A). For a topographic image represented by a height $h(x,y)$, where $x$ and $y$ are spatial coordinates (unit: pixel), the correction is performed sequentially. First, CellMAP reads the height at the origin, i.e.*,* at the bottom-left of the image ($x=1$; $y=1$), and rescales the map with respect to this value to obtain the rescaled height ${h}_{0}$ for each pixel:

1 $$h_{0} \left( {x,y} \right) = h\left( {x,y} \right) - h\left( {1,1} \right)$$

Second, CellMAP fits the tilted substrate by a plane, mathematically described by:

2 $$h_{s} \left( {x,y} \right) = a\left( {x - 1} \right) + b\left( {y - 1} \right)$$

where $a$ and $b$ are the slopes of the tilted surface in the orthogonal directions $x$ and $y$, respectively. By default, CellMAP performs two successive linear fits: along the first column ${h}_{0}(x,1)$ and along the first line ${h}_{0}(1,y)$ to estimate the coefficients $a$ and $b$, respectively. The corrected image is therefore given by:

3 $$h_{c} = h_{0} - h_{s}$$

where ${h}_{c}(x,y)$ is the corrected height at each pixel. Figure 3B displays the results of the flattening operation. The height histograms associated with the raw and leveled cell are shown in Fig. 3C. Although this default flattening operation is straightforward and generally leads to acceptable results, there are cases where it may fail (Fig. 3D, middle panel). Whenever the image shows any object in addition to the cell (e.g., see the pink circle for the Raw cell in Fig. 3D), another CellMAP function enables the user to select a region of interest (see ROI marked by a blue line in Fig. 3D) for tilt correction by fitting a new planar surface ${C}_{s}$ to this ROI, leading to a new $(a,b)$ pair (Fig. 3D, right panel). We strongly recommend checking the absence of tilt after “Flattening” by shrinking the color scale down to a small range of a few tens of nanometers; the substrate height should be flat, with its height fluctuating around zero (Fig. 3B and Fig. 3D, right panel).

#### Thresholding

Force mapping with high resolution implies the processing of many pixels and force curves. Typically, AFM images of cells obtained with the QI mode contain from $64 \times 64$ to $256 \times 256$ pixels and as many force curves. With thousands or tens of thousands of curves, the user cannot check the accuracy of each individual fitting operation performed on each curve. Hence, many pixels in the final image contain aberrant values of stiffness or height. They create local peak discontinuities within the map. To address this issue, the thresholding tool implemented in CellMAP enables the user to isolate and remove these outliers. The thresholding tool is also useful to focus on the height and stiffness of the cell, by separating the cell pixels from the substrate pixels.

**Fig. 4:**
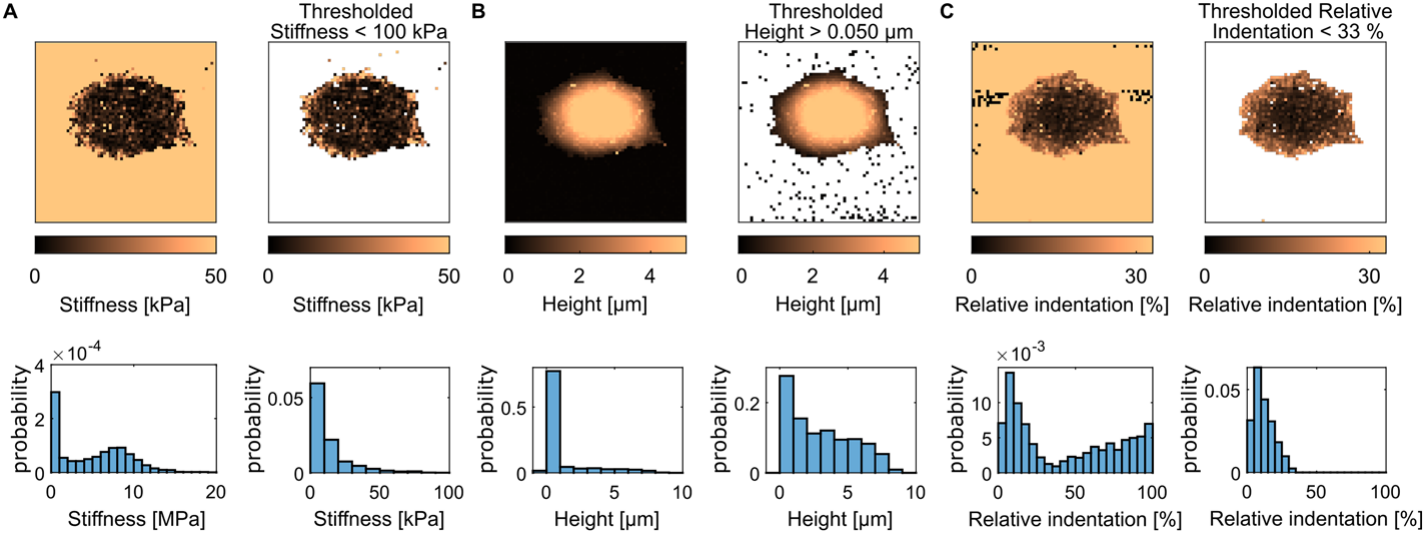
Thresholding of the maps of cell #1 (see Fig. 3B). Thresholding values are listed at the top of each panel. In each panel, the map or graph on the left and right represent the unthresholded and thresholded data, respectively. In maps, the white background represents the absence of values (NaNs). **A** Stiffness maps and corresponding distributions (at the top and bottom of the panel, respectively). **B** Height maps and corresponding distributions. **C** Relative indentation maps and corresponding distributions

The Thresholding tool sets aside pixels whose height and/or stiffness are out of a user-defined range. For example, in Fig. 4A, an upper bound threshold of $100 \text{kPa}$ is applied on the stiffness map $E(x,y)$ of a cell. Pixels whose stiffness is greater than this value are removed, not only from the stiffness map, but also from all the other maps, including the height map for example. Therefore, the pixels corresponding to the underlying substrate are removed from the map (top panels in Fig. 4A), and pixels whose stiffness is in the megapascal range (corresponding to the substrate surface) are now discarded (see the distribution of the stiffness in the bottom panels of Fig. 4A). Figure 4B shows a lower bound threshold of $50 \text{nm}$ applied on the contact point height map: $h(x,y)$ data points that are lower than $50 \text{nm}$ are omitted. Accordingly, the resulting histogram better reflects the distribution of cell height. Finally, Fig. 4C displays images resulting from applying an upper bound threshold on the local relative indentation ${\delta }_{r}(x,y)$:

4 $$\delta_{r} = \frac{{\delta_{\max } }}{{C_{c} }}$$

where ${C}_{c}$ is the tilt-corrected height and ${\delta }_{\text{max}}$ is the maximum indentation extracted from the force‒indentation curves (see Fig. 1A). This ratio quantifies how deep the AFM tip penetrates the cell with regard to the cell height, for each pixel. For instance, if for a certain pixel, the cell height is *h*_*c*_ = 10μm, and we apply an indentation of δ_max_ = 1 μm, then the relative indentation is δ_r_ = 10%. In summary, CellMAP enables the user to apply a unique threshold, as shown in Figs. 4A-C, or a combination of successive thresholds on any available map.

#### Filtering

CellMAP provides a variety of tools to remove unwanted objects on maps, such as isolated clusters of pixels, abnormal peaks and holes.

**Fig. 5:**
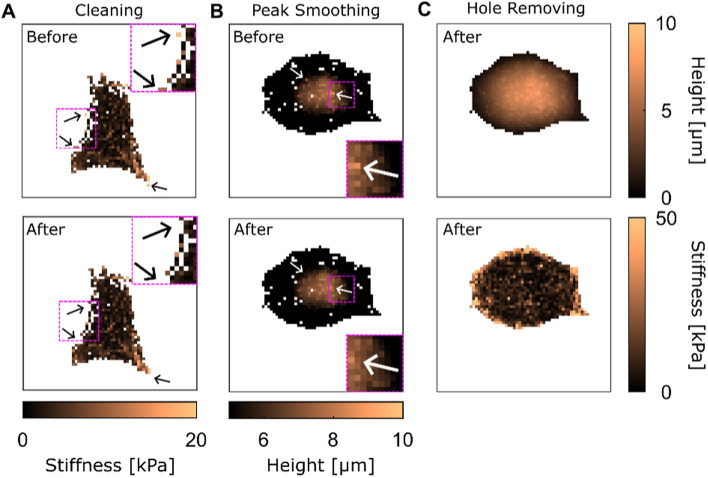
Filtering. In all panels, the white background represents the absence of values (NaNs). **A** Effect of the cleaning on the stiffness map. Black arrows indicate isolated clusters (top panel) that are removed by CellMAP (bottom panel). Insets display a zoom of the region of interest (ROI) delimited by a pink square. **B** Effect of peak smoothing on height map. White arrows indicate data points (top panel) that are smoothed by CellMAP (bottom panel). Insets display a zoom of the dashed ROI. **C** Effect of hole removing on height (top panel) and stiffness (bottom panel) maps. This step of the process follows the one obtained in the bottom graph of panel (**B**)

First, the “Cleaning” function removes isolated patches whose size is smaller than a user-defined input (specified as a number of pixels). Figure 5A displays the effect of “Cleaning”, where patches smaller than 50 pixels are removed (black arrows). If any unwanted item persists, we recommend gradually expanding the user-defined cleaning area by simply increasing the number of pixels in the Cleaning function.

Then, “Peak smoothing” is an upper bound threshold on the local variation within the considered map $M(x,y)$, and smooths, rather than removes, the data points (Fig. 5B). Here, $M$ represents either $h$, $E$ or ${\delta }_{r}$, hereafter called “the data”. A common situation in AFM data, where this option is particularly useful, concerns local artifacts on heights. These are caused by the inability to extract the height from some of the force curves collected during scanning, for example because a strong adhesion between the cell and the AFM tip affects the baseline of the force curve.

For every pixel, the peak smoothing function calculates the arithmetic mean of the data of its eight closest neighbors according to:

5 $$\overline{M}\left( {x,y} \right) = \frac{1}{8}\left( {\left[ {\mathop \sum \limits_{Y = y - 1}^{y + 1} M\left( {x - 1,Y} \right) + M\left( {x + 1,Y} \right)} \right] + M\left( {x,y - 1} \right) + M\left( {x,y + 1} \right)} \right)$$

Then, it lists the spatial coordinates $\{{x}_{m},{y}_{m}\}$ that verify the following inequality:

6 $$\left|M\left( {x_{m} ,y_{m} } \right) - \overline{M}\left( {x_{m} ,y_{m} } \right)\right| > \Delta$$

where ∆ is a user-defined threshold. Finally, the peak smoothing function replaces the original data with the mean $\overline{M }\left(x,y\right)$ in all the pixels for which the inequality in Eq. 6 is verified. In that case, we have:

7 $$M\left({x}_{m},{y}_{m}\right)= \overline{M }\left({x}_{m},{y}_{m}\right)$$

The process can be repeated multiple times until all the peaks are smoothed. Additionally, it may be helpful to calculate $\overline{M }$ manually on a specific peak by displaying data values via the “Data cursor” (a pointer used to select any pixel on the map) to estimate the appropriate value of ∆. Finally, all $\{{x}_{m},{y}_{m}\}$ pixels affected are removed from the other maps. For example, smoothing a height map with a threshold $\Delta$, leads to a list $\{{x}_{m},{y}_{m}\}$ of pixel coordinates where (i) height verifies inequality Eq. 6, (ii) height is replaced by Eq. 7, and (iii) stiffness data is substituted by a NaN.

Finally, as described above, filters may remove data points from the maps, which means that isolated empty pixels (numerically set to *NaN*) might be present (Fig. 5A, B). The function “Hole Removing” simply uses Eq. 5 to extrapolate data points, replacing these *NaN*s with *M* (Fig. 5C).

### Topographical calculations

#### Theory

From the heights obtained from the contact point maps, CellMAP also calculates both local curvature of cells and their global surface and volume. The “Curvature” function (available in the Add-ons menu) calculates gaussian and mean curvatures of a cell surface [24]. Briefly, the gaussian curvature is defined as $K={\kappa }_{1}{\kappa }_{2}$, where ${\kappa }_{1}$ and ${\kappa }_{2}$ are the principal curvatures at any given points. The mean curvature is defined as $H=({\kappa }_{1}+{\kappa }_{2})/2$. These two curvature datasets are displayed as two new maps.

Surfaces and volume are automatically calculated and displayed in the “Geometry” panel (Fig. 2). Specifically, two distinct areas are measured: the projected surface and the top area. The projected surface ${S}_{p}$ is the area of the cell in contact with the substrate:

8 $$S_{p} = l^{2} \times n$$

where $l$ is the pixel size in meter and $n$ is the number of data points remaining after the processing described above. Similarly, the volume of the cell is calculated as follows:

$$V = l^{2} \times \mathop \sum \limits_{{\left( {x,y} \right)}} C\left( {x,y} \right)$$

where $(x,y)$ is the data subset of non-*NaN*s data, which is thus a list of size $n$.

CellMAP also computes the surface area of cells from 3D morphology [25]. To do so, the $N\times N$ map is divided into $(N -1)\times (N -1)$ rectangles that are themselves split into two triangles. Introducing the cell spatial coordinates $\overrightarrow{u}(x,y) = \left(x;y;C(x,y)\right)$*,* the area of each triangle is defined as:

9 $$A\left(x,y\right)=\frac{1}{2} \left\Vert \left(\overrightarrow{u}\left(x+1,y\right)-\overrightarrow{u}\left(x,y\right)\right)\times \left(\overrightarrow{u}\left(x,y+1\right)-\overrightarrow{u}\left(x,y\right)\right)\right\Vert$$

The sum of these triangle areas ${S}_{t}= \sum A$ approximates the top area of the cell.

#### Validation

**Fig. 6:**
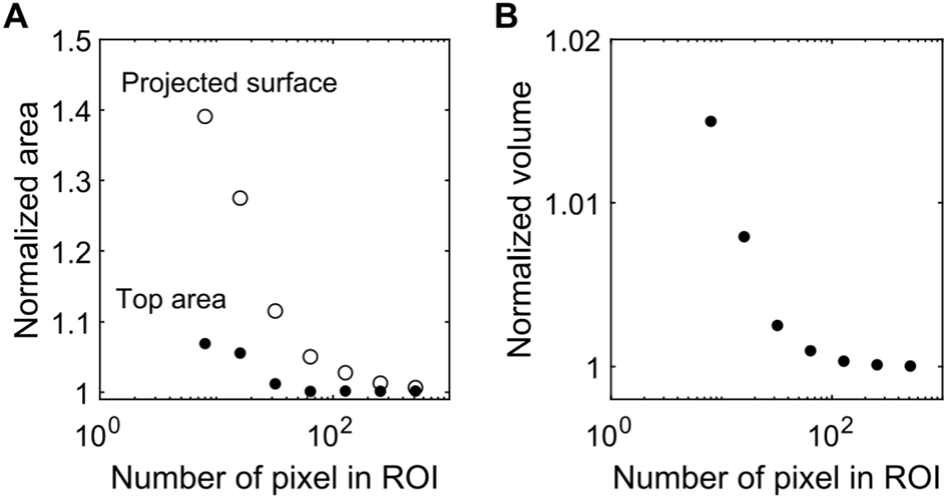
**A** Area (open circles: projected surface ${S}_{p}$; closed circles: top area ${S}_{t}$) and **B** volume $V$ for a computed hemisphere normalized by ${S}_{p} = \pi {R}^{2}$, ${S}_{t}=2 {R}^{2}$ and $V=\frac{2}{3}\pi {R}^{3}$, respectively. These quantities are displayed as a function of the number of pixels in the ROI or the image resolution

We demonstrate the reliability of CellMAP’s geometrical estimators across a broad range of image resolutions, using a simple geometrical object. A hemisphere of radius $R = 300 \mu m$ is numerically generated, centered on a $512\times 512$ array with a pixel length of 2 µm: the hemisphere measures 300 pixels in diameter. The theoretical values for areas and volume associated with this geometry, later used for normalization, are ${S}_{p} = \pi {R}^{2}$, ${S}_{t} = 2\pi {R}^{2}$ and $V=\frac{2}{3}\pi {R}^{3}$. To test how image resolution affects our estimates, we perform successive binning to generate images with $256\times 256$, $128\times 128$, …, and down to $8\times 8$ pixels. As shown in Fig. 6, image resolution is important for precision: increasing resolution decreases the error. The accuracy of the measurement performed by the CellMAP algorithm is also good, as the error is often below $10 \%$, except for ${S}_{p}$ at low resolution (below $32\times 32$ pixels).

**Fig. 7:**
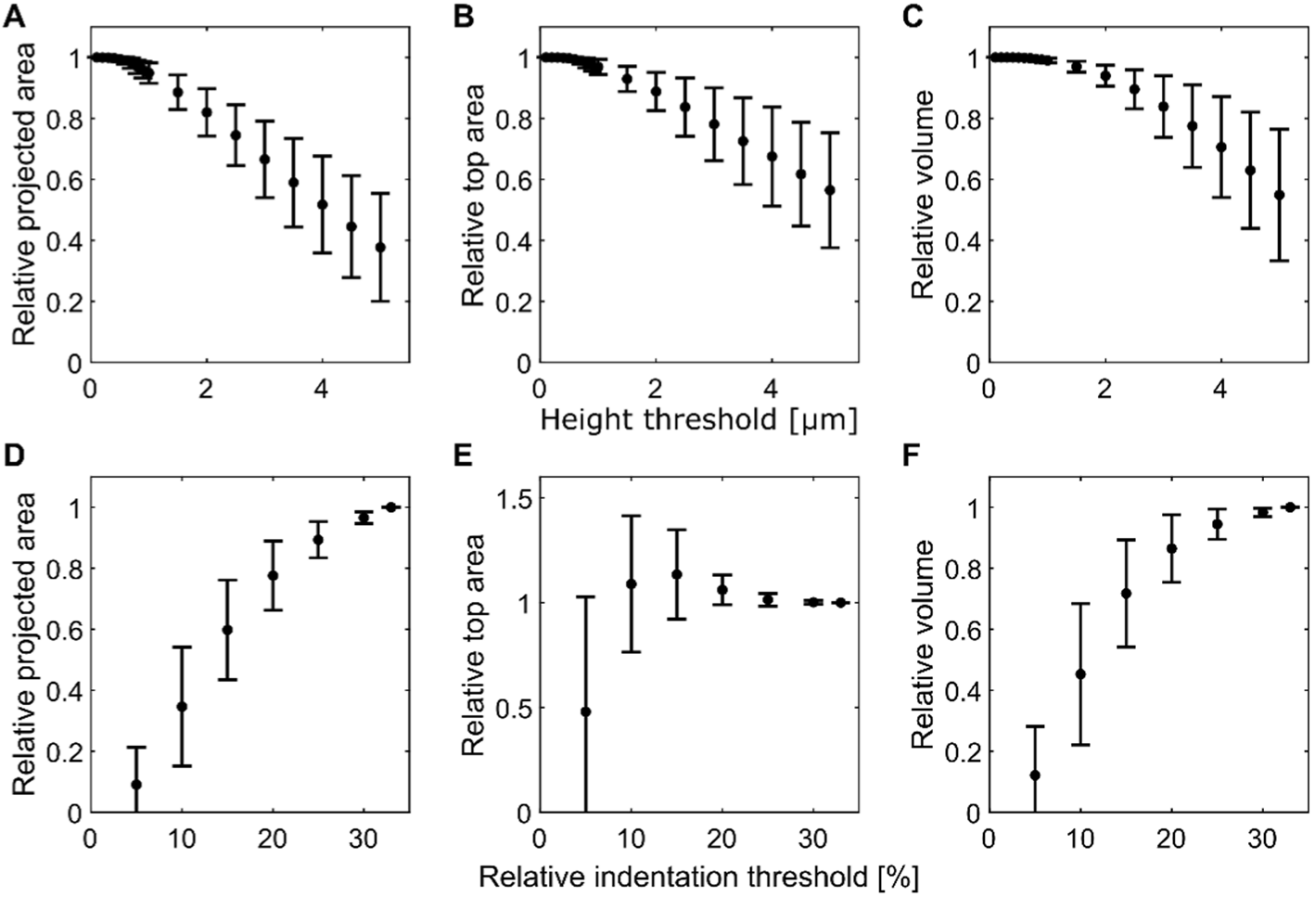
Effect of thresholds on geometrical estimators. Calculation are performed for N = 16 independent cells, and displayed as mean $\pm$ standard deviation. **A**–**C** The minimal height threshold is varied to study its impact on the projected surface $S_p$ (**A**), top area $A$ (**B**) and volume $V$ (**C**). These quantities are normalized by their values in the absence of height threshold. **D**–**F** The maximal relative indentation $\delta$ threshold is varied to study its impact on the projected surface $S_p$ (**D**), top area $A$ (**E**) and volume $V$ (**F**). These quantities are normalized by their values for a relative indentation threshold of 33%

**Fig. 8:**
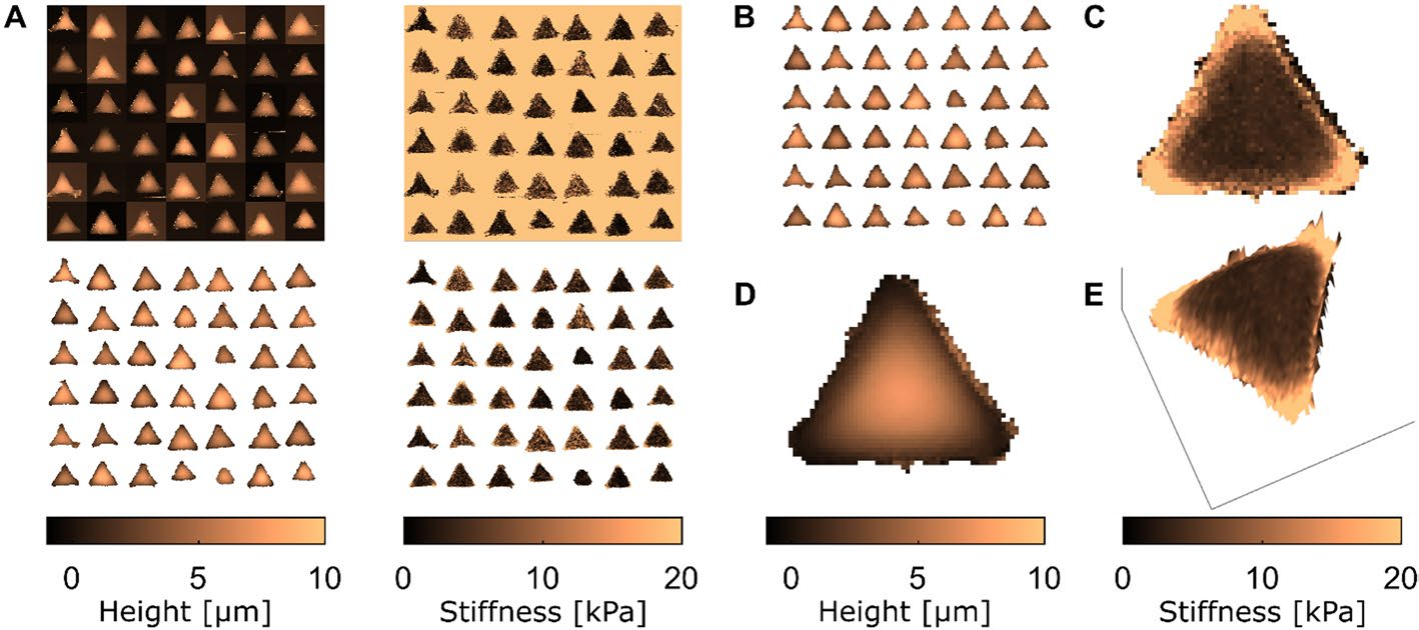
Making an “average cell”. **A** The top maps show raw representations of the height (left) and stiffness (right), whereas the bottom maps show representations after applying the workflow described in the text on 42 cells. **B** The 42 cells were centered with respect to their barycenter. **C** Stiffness map of the average cell. **D** Height map of the average cell. **E** Average cell in which the color code depicting the stiffness shown in (**C**) is overlaid on a 3D topographic representation of the height map in (**D**)

Thresholds also affect values of the geometrical estimators for volume and surfaces. We varied the height (Fig. 7A–C) and relative indentation (Fig. 7D–F) thresholds and calculated the projected area, top area and volume using CellMAP. As expected, a large threshold in height and a low threshold in relative indentation strongly impact the estimates of geometrical factors. Therefore, we show that it might be crucial to perform this measurement to validate a choice of threshold during processing, as done in Fig. 4. Altogether, we applied this careful analysis on a cell population presented in the next section (Fig. 8). By doing so, we validated our choice to apply three consecutive thresholds, by keeping data points > 0.1 µm in height, < 30 % in relative indentation and < 100 kPa in stiffness, guarantying the good convergence of the geometrical estimators.

### Cell statistics and display

After processing AFM images of single cells as described above, the user can combine all the measurements made on single cells into outputs reflecting the measurements made on a series of cells (see Fig. 1). The distribution of the height and stiffness of the cell population is displayed in the “Distribution” and “Statistics” panels (Fig. 2), which exhibit histograms and statistical parameters such as the mean, median, and standard deviation, among others. These data are easily exportable via CellMAP.

In addition, we developed a tool to visualize quickly the properties of a series of cells by computing the average topographical or mechanical maps of a cell population in which all the cells display a similar shape. In a previous study, we used it to compare the average properties of distinct cell populations [12]. Figure 8 shows an example of combining the quantitative measurements of single-cell processing made on 42 cells into a single “average cell”, using the function “Make Montage”. The spatial distributions of both height and stiffness of a series of cells are represented on a single image (see Fig. 8A). CellMAP generates the graphical representation of an average cell, first by using the “Centering” function that aligns the maps of all cells (see Fig. 8B). Each individual cell’s barycenter is translated toward the center of each map. Then, the “Averaging” function creates the average cell via the maps of all individual cells by calculating the arithmetic mean of height or stiffness at each position $(x,y)$ (Figs. 8C, D). This method enables the user to better exhibit specific features of the cell population: for instance, the average cell in Fig. 8C shows a higher stiffness at the three corners with respect to its center. Finally, the “Topography” function generates a 3-dimensional image of the selected cell, with a user-defined z-axis and a color code corresponding to the “Type of data” from the “Mapping” panel (Fig. 8E).

**Fig. 9:**
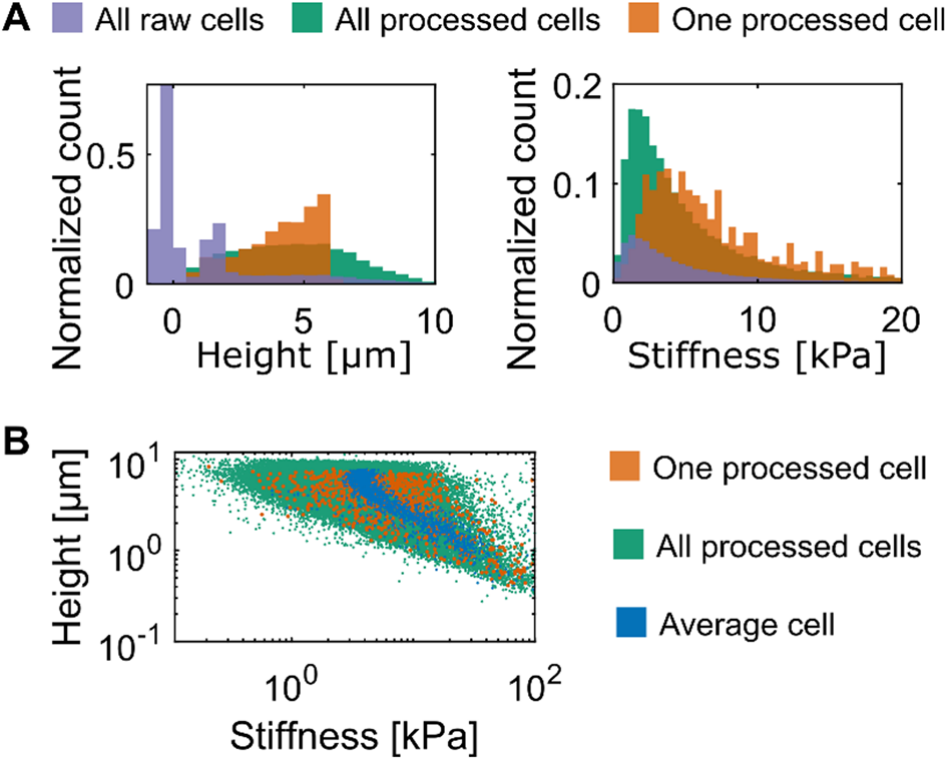
Comparing the analysis on one cell with the analysis on a population of cells. **A** Histograms of heights (left) and stiffness (right). These graphs depict the distributions of height and stiffness made on the cell population shown in Fig. 8A before (purple) and after (green) processing. The distributions shown in orange represent one of the 42 cells, randomly picked. **B** Height as a function of stiffness for one processed cell (orange) randomly picked, for the processed population (green) and for the average cell (blue) shown in Fig. 8C–E

The histograms in Fig. 9A highlight the strengths of CellMAP. Indeed, CellMAP displays the height and stiffness distributions of the whole cell population at each processing step (compare the distribution in purple with the distribution in green that represent raw and processed datasets, respectively). Whereas the heights of the glass substrate make the largest contribution to the histogram of the raw data, the histogram of the processed data pinpoints the heights of the cells. Similarly, many pixels of the raw stiffness maps are greater than tens of kilopascals (as they reflect the stiffness of the glass substrate) and are discarded in the processed maps. Furthermore, CellMAP can capture the height and stiffness of a cell population; those might dramatically differ from the height and stiffness of one random cell isolated from that same population (compare the distribution in orange with the distribution in green, Fig. 9A).

Finally, the Add-on called “Plot Y=f(X)” opens a new window to display one “Type of data” (e.g., height) as a function of another one (such as stiffness, see Fig. 9B). That might be useful for identifying potential correlations between different parameters. Again, there are quantitative differences between the cell population (green dots in Fig. 9B), one randomly picked cell (orange dots) and the average cell shown in Fig. 8C–E (blue dots in Fig. 9B). In summary, CellMAP enables the users to optimize their analyses of both single cells and cell populations.

## Conclusions

AFM is a unique technique as it can provide a nanoscale view of the mechanics and morphology of cells, which helps understanding the internal functioning of cells in both health and disease. However, gathering large datasets and performing statistical analyzes on cell populations from multiple AFM images has remained difficult. To better address this challenge, we present the CellMAP software tool. We designed CellMAP specifically to process batches of AFM images of living cells. CellMAP is the ideal counterpart of other open-source software tools that excel in analyzing individual cells: CellMAP offers new functions to handle statistical analyses of whole cell populations, providing a user-friendly interface for measuring the morphology and stiffness of cell populations. In addition, this article details the key steps of single-cell processing by CellMAP and CellMAP’s ability to clean individual images effectively, therefore optimizing the analytical process and making CellMAP a comprehensive package for processing AFM images.

To test the performance of CellMAP, we used it to calculate the surfaces and volumes of simulated shapes of known sizes (Figs. 6 and 7). Then, we used it to analyze a series of cells exhibiting a similar shape. In this case, CellMAP is useful to visualize the average characteristics of the cell population through the creation of an average cell. As shown in Fig. 9, the cells differ from each other, emphasizing the interest in studying both average cell characteristics and the variability among individual cells. For example, in cancer research, understanding the heterogeneity in mechanical properties among tumor cells can provide insights into metastatic potential [13, 26]. This highlights the utility of CellMAP for studying the biomechanics of living cells with statistical measurements of the height and stiffness performed over a large number of standardized cells.

As it is open-source, CellMAP is intrinsically suitable to improvement by new users. While it has specifically been designed and optimized for AFM images, CellMAP loads 2-dimensional matrices, which is the format of any image coming from other techniques such as widefield or confocal microscopy. Hence the cleaning and thresholding operations as well as the statistical processing of any quantity that is not necessarily a height or a stiffness is possible with CellMAP.

However, while CellMAP is effective for batch-processing AFM data maps from single-cell populations, it may face limitations when dealing with specific types of data and experimental conditions. For example, CellMAP is not optimized for directly processing raw force-distance curves in large-scale datasets. While it can handle pre-processed force-volume maps efficiently, users working with raw individual force curves are encouraged to perform preprocessing steps externally before utilizing CellMAP for further analysis. Additionally, analyzing datasets from continuous tissue samples, such as epithelial tissues or confluent cell layers, remains a challenge. Unlike single-cell datasets where distinguishing cells from the underlying substrate is straightforward, tissues often present complex variability in mechanical properties and less-defined cell boundaries. These factors may complexify statistical analysis and require additional methods for handling variability.

Potential directions for future iterations of CellMAP include adding functionalities to better process raw force-distance curves and address tissue sample heterogeneity. One promising direction is the use of machine learning algorithms for either pattern recognition in images or fitting of the force curves. The integration of automated preprocessing, analysis, and interpretation would significantly reduce the manual workload, enhancing the efficiency of CellMAP.

## Material and methods

### Cell culture

The MDA-MB-231 cells were cultured in Leibovitz’s L-15 + GlutaMAX medium supplemented with 10% fetal bovine serum at 37 °C. For AFM experiments on non-constrained cells, we coat Mattek Petri dishes (P35G-0-10-C) with 5 µg/cm^2^ fibronectin. The dishes were treated with UV light for 15 minutes and then incubated for one hour in a fibronectin solution in PBS. We then seeded the dishes with the cells and allowed them to adhere for 12 hours. For cells that adhered to fibronectin-coated micropatterns, we utilized CYTOO “Starter Chips” slides with medium-sized Y-shaped patterns. We fixed the slide in a Petri dish and incubated it for 30 minutes in culture medium at 37 °C. Then, we seeded the slides with 120,000 cells and allowed them to adhere for 12 hours.

### AFM imaging and force spectroscopy

For all the experiments, we used a JPK/Bruker Nanowizard 4 in QI mode to image living cells as described in previous studies [10, 12]. The QI mode is a fast force-mapping mode that collects force‒distance curves over a given scanning area, using a predefined ramp size, setpoint force, and scanning speed. After scanning, the force‒distance curves are batch-processed and undergo a series of processing operations, essentially to flatten the baseline and fit a contact point model to the force‒indentation part of the force curve to derive a stiffness [12]. The fit also provides the altitude of the contact point, that is, the vertical distance from the substrate at which the AFM tip hits the cell surface.

## Availability and requirements

Project name: CellMAP.

Project home page: https://sourceforge.net/projects/cellmap-afm/files/.

Operating system: Platform independent.

Programming language: Matlab.

Other requirements: MATLAB Runtime R2020b or higher (for standalone installation) or MATLAB R2020b or higher with the following toolboxes: Curve Fitting, Image Processing (11.4 or higher), Statistics and Machine Learning.

License: CC BY-NC-SA 4.0.

Any restrictions to use by non-academics: You may not use the material for commercial purposes.

## Data Availability

The datasets analyzed and generated and during the current study are available in the sourceforge repository [23], https://sourceforge.net/projects/cellmap-afm/files/
